# Second primary breast cancer after unilateral mastectomy alone or with contralateral prophylactic mastectomy

**DOI:** 10.1002/cam4.3394

**Published:** 2020-09-12

**Authors:** Shailesh Agarwal, Lisa Pappas, Cindy B. Matsen, Jayant P. Agarwal

**Affiliations:** ^1^ Department of Surgery University of Chicago Medical Center Chicago IL USA; ^2^ Huntsman Cancer Institute University of Utah Salt Lake City UT USA; ^3^ Department of Surgery University of Utah Salt Lake City UT USA

**Keywords:** breast cancer, contralateral prophylactic mastectomy, CPM, SEER, survival, unilateral mastectomy

## Abstract

**Background:**

An increasing number of patients undergo contralateral prophylactic mastectomy (CPM) for unilateral breast cancer. However, the benefit of CPM has not been quantified in the setting of contemporary breast cancer therapy.

**Methods:**

We performed an analysis of 180 068 patients in the Surveillance, Epidemiology, and End Results (SEER) database, diagnosed with unilateral ductal breast carcinoma between 1998 and 2013 and treated with unilateral mastectomy (UM) or CPM. UM was performed in 146 213 patients (81.2%); CPM was performed in 33 855 patients (19.8%). Primary outcome of interest was cumulative incidence of a second primary breast cancer in the ipsilateral or contralateral breast greater than 3 months after initial diagnosis. Cumulative incidence analysis was based on a Cox proportional model to generate curves of second primary breast cancer in any breast, ipsilateral breast only, or contralateral breast only.

**Results:**

Patients who underwent CPM had a significantly reduced incidence of second primary breast cancer 10 and 15 years after surgery (CPM 0.93% [0.73%, 1.12%] vs UM 4.44% [4.28%, 4.60%]). Patients who underwent CPM had significantly lower adjusted hazard of second primary breast cancer when compared with UM (HR 0.38 vs 1.0, *P* < .0001).

**Conclusions:**

CPM offers some protection from a second primary breast cancer, attributable to a reduced incidence in the contralateral breast. These findings provide additional information to providers and patients as they make decisions regarding surgical management. They should also be interpreted in the context of the absolute incidence of second primary breast cancer after UM and previous literature demonstrating no survival benefit.

## Graphical abstract

We examine second primary breast cancer in the setting of unilateral or bilateral mastectomy using a population‐level database. Our results show that although patients with bilateral mastectomy have reduced odds of second primary breast cancer, this must be interpreted in the context of an overall low rate of second primary breast cancer.

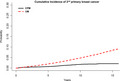

## 1 INTRODUCTION

Contralateral prophylactic mastectomy (CPM) is often performed for patients with unilateral breast cancer who are at high risk for a second primary breast cancer, including those with genetic predisposition or strong family history.^1^, ^2^, ^3^ Patients with BRCA1 or BRCA2 mutations have up to a 40% cumulative risk of contralateral breast cancer (CBC) 20 years after initial diagnosis.^1^ However, the past 15 years have seen increasing utilization of CPM in low‐risk patients with unilateral breast cancer; fear of a second primary breast cancer drives the desire for CPM, even among patients at low risk for CBC.^4^, ^5^ To facilitate effective shared decision making with respect to CPM, there is a need for contemporary population‐level data on the incidence of any second primary breast cancer or CBC,^6^ especially as dissemination of information to the patient is recognized as a contributor to decision making and risk assessment.^7^, ^8^

Population‐based studies have shown that, in the absence of a predisposition toward developing breast cancer, patients who undergo CPM do not experience improved survival.^9^, ^10^, ^11^, ^12^, ^13^ In addition, patients who undergo CPM are at risk for longer operative and hospitalization times, and those who undergo subsequent reconstruction are at a further higher risk for complications related to the reconstructive course.^14^ Furthermore, patients who undergo CPM experience increased medical costs with similar quality of life when compared with patients who undergo unilateral mastectomy (UM) and subsequent surveillance.^15^ Therefore, it is imperative that information relating the risk of a CBC be conveyed to patients who are considering a CPM.

There are limited data on the incidence of a second primary breast cancer in patients who have had mastectomy for breast cancer^16^, ^17^; this is even less well understood in patients who have had CPM, especially on a population level.^6^, ^18^, ^19^ Small studies with limited cohorts have demonstrated varying impact of CPM on CBC; however, these studies have also demonstrated improved survival after CPM^18^, ^19^—a finding which is now disputed through larger population‐level studies.^9^, ^10^, ^11^, ^12^, ^13^ Population‐level information regarding the risk of a second primary breast cancer with UM or CPM would help patients and physicians make informed decisions regarding the benefit of CPM, especially when taking into consideration that studies have demonstrated no improved survival with CPM. In this study we use population‐level data from the Surveillance, Epidemiology, and End Results (SEER) database to determine the incidence and hazard ratio of a second primary breast cancer among patients treated with UM or CPM.

## 2 METHODS

### 2.1 Data source

Population‐level de‐identified data were extracted from the SEER cancer database for patients with a primary diagnosis of ductal carcinoma of the breast during the years 1998 through 2013 (Surveillance, Epidemiology, and End Results (SEER) Program SEER*Stat Database: Incidence—SEER 18 Regs Research Data + Hurricane Katrina Impacted Louisiana Cases, Nov 2015 Sub (1973‐2013 varying);—Linked To County Attributes—Total US, 1969‐2014 Counties, National Cancer Institute, DCCPS, Surveillance Research Program, released April 2016, based on November 2015 submission). The SEER database collects patient‐level data for all index malignant tumors in 19 cancer registries across the United States and has been reported to capture up to 28% of the nation's population.^20^ This database is regarded as nationally representative and contains detailed demographic, socioeconomic, oncologic, and therapeutic information. To ensure data accuracy, chart abstracters undergo extensive training. Malignant tumors are encoded by use of the ninth revision of the International Classification of Diseases for Oncology.

### 2.2 Patient inclusion/exclusion

Female patients with a new primary diagnosis of unilateral breast cancer between 1998 and 2013 were included in this analysis. Patients were included regardless of American Joint Committee on Cancer (AJCC) tumor stage (including stages 0‐IV). All patients had initial primary histologic diagnosis of ductal carcinoma (8500) and received either unilateral mastectomy alone (UM) (surgery codes 41, 43‐46, 51, 53‐56) or unilateral mastectomy with contralateral prophylactic mastectomy (CPM) (surgery codes 42, 47‐49, 52, 57‐59, 63, 75).

### 2.3 Statistical analysis

Patients were divided into two categories—those receiving unilateral mastectomy alone (UM) or unilateral mastectomy with contralateral prophylactic mastectomy (CPM). Patients were determined to have a diagnosis of a second primary breast cancer if they had a subsequent diagnosis of primary breast cancer in the ipsilateral or contralateral breast greater than 3 months after the initial primary breast cancer. Cumulative incidence analysis was based on a Cox proportional model to generate curves of second primary breast cancer in breast, ipsilateral breast only, or contralateral breast only were plotted. Kaplan‐Meier analysis was used to generate point estimates for 10‐ and 15‐year cumulative rates.

Time‐to‐event methods were used to analyze the outcome, with time calculated as the months from the date of first breast cancer to the earliest of the following events: second primary breast cancer diagnosis, death, loss of follow‐up, or administrative cut‐off; for time to second primary breast cancer in the contralateral breast, events were censored at the time of a second ipsilateral primary breast cancer. The proportion with second primary breast cancer diagnosis, accounting for the competing risk of death, was estimated using cumulative incidence estimation (Figure S1). Fine and Grey competing risk regression was used for all the models. Multivariate model selection compared a base model adjusting only for mastectomy type (UM or CPM), then adding demographic information, and then oncologic information. Models were compared using the maximum likelihood method and comparing Bayesian information criterion (BIC) values. All analyses were performed using R (ver. 3.3.2)[R Core Team, 2013] and packages cmprsk for analysis, Gmisc for plot and table output, and knitr for reproducible research.

## 3 RESULTS

### 3.1 Population characteristics

A total of 180 068 patients (age range 15‐105 years, mean 57.8 years (SD 14.3 years)) who underwent mastectomy for a diagnosis of unilateral breast ductal carcinoma were included in this study. Unilateral mastectomy alone (UM) was performed in 146 213 patients (81.2%) while unilateral mastectomy with contralateral prophylactic mastectomy (CPM) was performed in 33 855 patients (19.8%) (Table 1). CPM patients tended to be younger when compared with UM patients (50 years vs 60 years, *P* < .0001). In addition, population composition by race was different; a higher proportion of CPM patients were White when compared with UM patients (86% vs 77%, *P* < .001) (Table 1).

**TABLE 1:** Population demographic and oncologic characteristics at time of first primary breast cancer diagnosis

	Bilateral (n = 33 855)	Unilateral (n = 146 213)	*P*‐value
Race			
White	28 992 (86%)	112 745 (77%)	<.0001
Black	2528 (7%)	16 971 (12%)	
Other	2176 (6%)	15 936 (11%)	
Unknown	159 (0%)	561 (0%)	
Age at Diagnosis	50 (± 12)	60 (± 14)	<.0001
Grade			<.0001
Well differentiated; Grade I	4319 (13%)	17 804 (12%)	
Moderately differentiated; Grade II	12 347 (36%)	54 577 (37%)	
Poorly differentiated; Grade III	14 939 (44%)	64 345 (44%)	
Undifferentiated; anaplastic; Grade IV	633 (2%)	3344 (2%)	
Unknown	1617 (5%)	6143 (4%)	
Stage			<.0001
0	3126 (9%)	9620 (7%)	
I	11 906 (35%)	44 866 (31%)	
II	12 869 (38%)	56 846 (39%)	
III	5420 (16%)	30 952 (21%)	
IV	534 (2%)	3929 (3%)	
ER Status			<.0001
Positive	23 223 (69%)	95 362 (65%)	
Negative	8366 (25%)	35145 (24%)	
Borderline	58 (0%)	300 (0%)	
Unknown	2208 (7%)	15 406 (11%)	
PR status			<.0001
Positive	20 037 (59%)	79 237 (54%)	
Negative	11 249 (33%)	49 219 (34%)	
Borderline	118 (0%)	754 (1%)	
Unknown	2451 (7%)	17 003 (12%)	
Tumor size			<.0001
0‐2	15 585 (46%)	57 235 (39%)	
>2‐4	10 684 (32%)	50 845 (35%)	
>4	4889 (14%)	26 862 (18%)	
Unknown	2697 (8%)	11 271 (8%)	
Node group			<.0001
0 nodes	19 475 (58%)	74 816 (51%)	
1‐3 nodes	8504 (25%)	37 793 (26%)	
>3 nodes	5876 (17%)	33 604 (23%)	
Time (m) follow‐up	54 (± 43)	73 (± 50)	<.0001

### 3.2 Second primary breast cancer incidence

**FIGURE 1:**
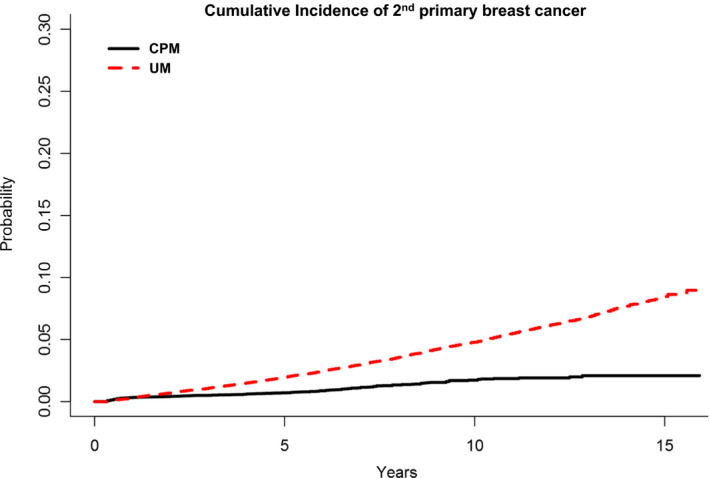
Kaplan‐Meier cumulative rate estimates of any secondary primary breast cancer among CPM patients (n = 33 855) when compared with UM patients (n = 146 213) (10‐year: 0.93% [0.73%, 1.12%] vs 4.44% [4.28%, 4.60%]; 15‐year: 1.15% [0.84%, 1.46%] vs 7.77% [7.36%, 8.18%])

**FIGURE 2:**
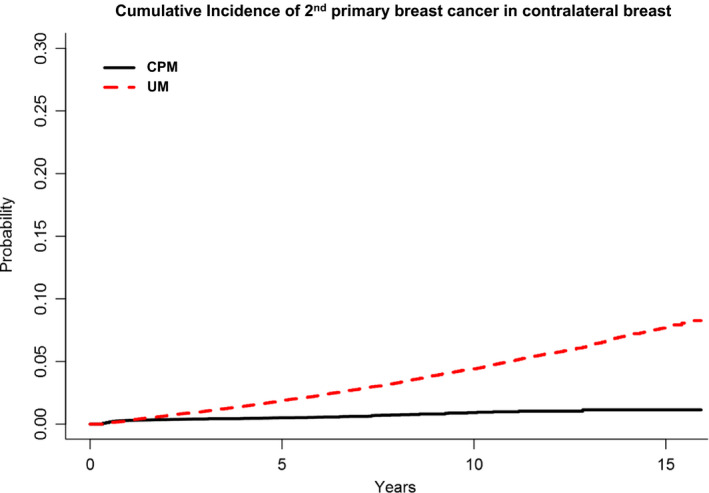
Kaplan‐Meier cumulative rate estimates of secondary primary breast cancer in the contralateral breast among CPM patients (n = 33 855) when compared with UM patients (n = 146 213)

**FIGURE 3:**
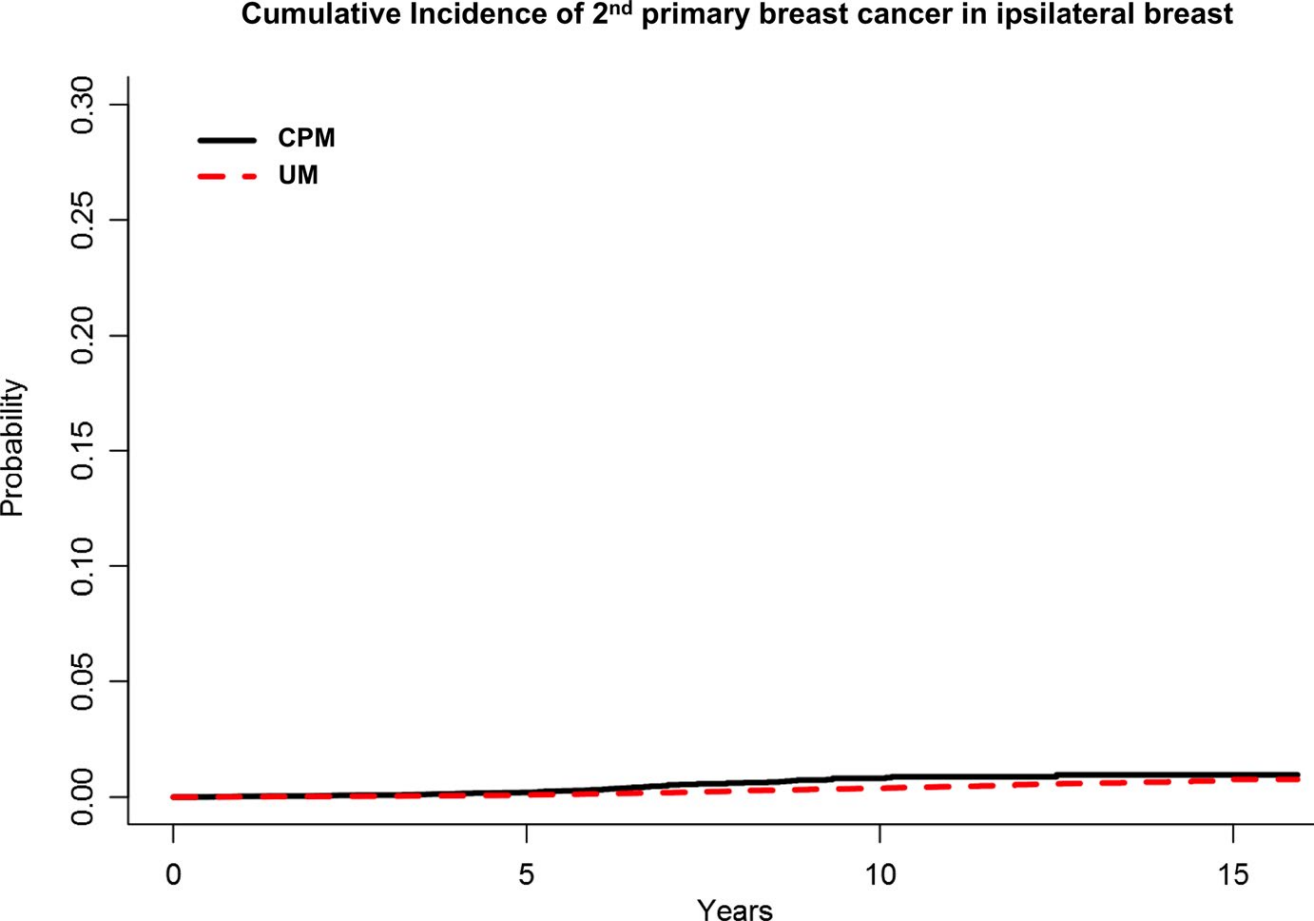
Kaplan‐Meier cumulative rate estimates of secondary primary breast cancer in the ipsilateral breast among CPM patients (n = 33 855) when compared with UM patients (n = 146 213)

The 10‐ and 15‐year Kaplan‐Meier cumulative rate estimates of a second primary breast cancer was substantially lower among CPM patients when compared with UM patients (10‐year: 0.93% [0.73%, 1.12%] vs 4.44% [4.28%, 4.60%]; 15‐year: 1.15% [0.84%, 1.46%] vs 7.77% [7.36%, 8.18%]) (Figure 1). This was attributable to a reduced cumulative incidence of second primary breast cancer in the contralateral breast among CPM patients when compared with UM patients (Figure 2); a similarly substantial reduction was not observed in the cumulative incidence of second primary breast cancer in the ipsilateral breast (Figure 3).

Among those with a second primary breast cancer, CPM patients had a significantly reduced proportion of second primary breast cancer in the contralateral breast when compared with UM patients (65% vs 93%, *χ* ^2^ = 221, *P* < .0001).

The mean time to diagnosis of second primary breast cancer was 35 months in CPM patients and 55 months in UM patients (Table 2); the mean size of the second primary breast cancer at time of diagnosis was similar (4 mm) between the two cohorts (Table 2). Average annual incidence of a second primary breast cancer was higher in the contralateral breast for patients who underwent UM (0.38%) versus CPM (0.04%) (Table 3).

**TABLE 2:** Demographic and oncologic characteristics of patients with second primary breast cancer diagnosis

	Bilateral (n = 243)	Unilateral (n = 4071)
Race		
White	197 (81%)	3117 (77%)
Black	29 (12%)	533 (13%)
Other	17 (7%)	417 (10%)
Unknown	0 (0%)	4 (0%)
Age at first Diagnosis	50 (± 12)	58 (± 13)
Site of 2nd diagnosis		
Contralateral	158 (65%)	3778 (93%)
Ipsilateral	85 (35%)	293 (7%)
Histology		
Different	95 (39%)	1565 (38%)
Same (8500)	148 (61%)	2506 (62%)
Tumor stage		
0	76 (31%)	1075 (26%)
I	75 (31%)	1415 (35%)
II	36 (15%)	738 (18%)
III	22 (9%)	385 (9%)
IV	11 (5%)	182 (4%)
Unknown	23 (9%)	276 (7%)
ER Status of 2nd diagnosis		
Borderline	0 (0%)	6 (0%)
Negative	49 (20%)	903 (22%)
Positive	122 (50%)	2292 (56%)
Unknown	72 (30%)	870 (21%)
PR Status of 2nd diagnosis		
Borderline	0 (0%)	23 (1%)
Negative	84 (35%)	1426 (35%)
Positive	84 (35%)	1698 (42%)
Unknown	75 (31%)	924 (23%)
Tumor size of 2nd diagnosis (mm)	4 (SD 1)	4 (SD 1)
Time (m) to 2nd diagnosis	35 (SD 36)	55 (SD 41)

**TABLE 3:** Annual at risk and second primary breast cancer incidence

Years postmastectomy	CPM					UM				
	# at risk	# new ipsilateral event	% new ipsilateral event	# new contralateral event	% new contralateral event	# at risk	# new ipsilateral event	% new ipsilateral event	# new contralateral event	% new contralateral event
1	27 718	7	0.03%	17	0.06%	130 294	21	0.02%	683	0.52%
2	22 932	8	0.03%	9	0.04%	114 987	25	0.02%	460	0.40%
3	18 759	9	0.05%	5	0.03%	100 719	24	0.02%	390	0.39%
4	15 210	8	0.05%	8	0.05%	87 765	16	0.02%	326	0.37%
5	11 924	11	0.09%	4	0.03%	75 723	30	0.04%	282	0.37%
6	9271	15	0.16%	4	0.04%	64 797	32	0.05%	258	0.40%
7	7159	8	0.11%	5	0.07%	54 777	34	0.06%	218	0.40%
8	5595	4	0.07%	2	0.04%	45 967	29	0.06%	201	0.44%
9	4363	2	0.05%	4	0.09%	37 934	16	0.04%	149	0.39%
10	3267	1	0.03%	1	0.03%	30 188	17	0.06%	136	0.45%
11	2360	1	0.04%	0	0.00%	23 282	11	0.05%	82	0.35%
12	1589	1	0.06%	1	0.06%	16 883	8	0.05%	63	0.37%
13	895	0	0.00%	0	0.00%	10 698	2	0.02%	47	0.44%
14	363	0	0.00%	0	0.00%	5029	2	0.04%	13	0.26%
15	148	0	0.00%	0	0.00%	2324	0	0.00%	5	0.22%
Annual average			0.05%		0.04%			0.04%		0.38%

### 3.3 Hazard ratio of second primary breast cancer

Multivariate logistic regression was performed to determine the hazard of a second primary breast cancer while accounting for demographic and oncologic factors. CPM patients had a significantly lower hazard of second primary breast cancer when compared with UM (HR 0.38 vs 1.0, *P* < .0001) (Table 4). Furthermore, CPM patients had a significantly lower hazard of second primary breast cancer in the contralateral breast when compared with UM patients (HR 0.27 vs 1.0, *P* < .0001) (Table 5).

**TABLE 4:** Unadjusted hazard ratio of second primary breast cancer occurring in contralateral breast

	HR	95% CI	*P*‐value
Mastectomy type			
UM	1.00	—	—
CPM	0.38	0.33, 0.44	<.0001
Age (y)	0.98	0.98, 0.99	<.0001
Race			
White	1.00	—	—
Black	1.14	1.02, 1.27	<.05
Other	1.09	0.97, 1.22	.1400
Marital status			
Married	1.00	—	—
Unmarried	0.90	0.83, 0.97	<.01
Tumor stage			
0	1.63	1.41, 1.89	<.0001
I	1.00	—	—
II	0.85	0.74, 0.97	<.05
III	0.61	0.51, 0.74	<.0001
IV	0.32	0.21, 0.49	<.0001
Tumor grade			
I	1.00	—	—
II	0.82	0.73, 0.91	<.001
III	0.74	0.65, 0.83	<.0001
IV	0.92	0.75, 1.13	.4400
Unknown	0.74	0.61, 0.89	<.01
ER status			
ER Negative	1.00	—	—
ER Positive	0.82	0.73, 0.92	<.001
ER Borderline	1.34	0.75, 2.41	.3200
ER Unknown	0.61	0.46, 0.80	<.001
PR status			
PR Negative	1.00	—	—
PR Positive	1.01	0.90, 1.13	.8700
PR Borderline	1.04	0.66, 1.64	.8600
PR Unknown	1.28	0.99, 1.66	.0590
Nodal status			
0 nodes	1.00	—	—
1‐3 nodes	0.86	0.77, 0.97	<0.05
>3 nodes	0.87	0.76, 0.99	<0.05
Tumor size (cm)			
<2 cm	1.00	—	—
2‐4 cm	0.90	0.81, 1.01	.0690
>4 cm	1.09	0.95, 1.25	.2000
Unknown	1.15	1.00, 1.32	<.05

**TABLE 5:** Adjusted hazard ratio of second primary breast cancer occurring in contralateral breast

	HR	95% CI	*P*‐value
Mastectomy type			
UM	1.00	—	—
CPM	0.27	0.23, 0.32	<.0001
Age (y)	0.99	0.98, 0.99	<.0001
Race			
White	1.00	—	—
Black	1.07	0.95, 1.20	.2900
Other	1.09	0.96, 1.22	.1700
Unknown	0.32	0.10, 1.01	.0520
Marital status			
Married	1.00	—	—
Unmarried	0.92	0.85, 0.99	<.05
Tumor stage			
0	1.47	1.25, 1.73	<.0001
I	1.00	—	—
II	0.90	0.78, 1.03	.1300
III	0.70	0.58, 0.85	<.001
IV	0.33	0.22, 0.52	<.0001
Tumor grade			
I	1.00	—	—
II	0.79	0.71, 0.89	<.001
III	0.72	0.64, 0.82	<.0001
IV	0.92	0.74, 1.15	.4600
Unknown	0.68	0.56, 0.83	<.001
ER status			
ER Negative	1.00	—	—
ER Positive	0.77	0.68, 0.87	<.0001
ER Borderline	1.24	0.67, 2.31	.5000
ER Unknown	0.63	0.46, 0.85	<.01
PR status			
PR Negative	1.00	—	—
PR Positive	1.02	0.92,1.15	.7000
PR Borderline	0.93	0.57, 1.52	.7700
PR Unknown	1.19	0.89,1.57	.2400
Nodal status			
0 nodes	1.00	—	—
1‐3 nodes	0.84	0.75, 0.95	<.01
>3 nodes	0.82	0.71, 0.95	<.01
Tumor size (cm)			
<2 cm	1.00	—	—
2‐4 cm	0.90	0.81, 1.01	.0840
>4 cm	1.05	0.92, 1.21	.4700
Unknown	1.13	0.97, 1.31	.1200

## 4 DISCUSSION

Previous studies have demonstrated that, among patients without a clear predisposition toward developing breast cancer, contralateral prophylactic mastectomy (CPM) does not improve overall or cancer‐specific survival when compared with unilateral mastectomy.^9^, ^10^, ^11^, ^12^, ^13^ Despite these findings, rates of CPM continue to increase.^12^, ^13^, ^21^, ^22^, ^23^, ^24^, ^25^ Although the reasons for this trend are under investigation,^2^, ^5^, ^7^, ^21^, ^22^, ^26^, ^27^, ^28^ patient anxiety and overestimation of personal contralateral breast cancer risk (CBC) have received substantial attention as a driving force for the increasing utilization of CPM.^4^, ^15^, ^21^, ^24^, ^27^, ^29^ Using patient‐directed surveys, one previous study has shown that patients overestimate their risk for CBC, with 10‐year self‐reported risk estimates over 30%.^29^ However, these self‐reported risk assessments contrast sharply with several population‐based studies—Reiner et al reported the 10‐year cumulative risk of contralateral breast cancer (CBC) to range from 4.0% to 7.0% among patients with no family history of breast cancer or *BRCA1/2* mutation.^17^ Even among patients with a family history of bilateral breast cancer, they reported the 10‐year cumulative risk of CBC to range from 13% to 24%.^17^ However, Reiner et al included only a limited cohort of 1700 patients, and did not directly compare UM with CPM; or did they provide a population breakdown of patients undergoing UM or CPM. Gao et al reported the 10‐year incidence of CBC to be 6.1% using the SEER database (1973 through 1996) with over 130 000 patients.^16^ Similar to Reiner et al, Gao et al did not compare UM with CPM. Using the SEER database, we found the cumulative incidence of CBC among patients with UM to be 4.44% (95% CI [4.28%, 4.60%]). In contrast to the findings reported by Gao et al, our study includes a more contemporary cohort of patients. This is particularly critical, as breast cancer therapy has evolved with improvements in adjuvant radiation and chemotherapy, as well as recommendations for antiestrogen therapy.^30^, ^31^, ^32^, ^33^ Our study also includes a broader regional representation due to the expansion of SEER registry sites since 2000. In addition, Gao et al did not adjust for tumor stage, a major determinant of systemic therapy (eg, chemotherapy) which may in turn impact the risk of CBC; or did they adjust for first primary breast cancer ER status, which influences the use of systemic antiestrogen therapy.^30^, ^34^

We next determined the hazard of CBC among patients who undergo CPM, relative to UM. One prior study by Herrinton et al used a limited cohort of patients diagnosed with breast cancer from 1979 through 1999 (n = 1400) and found the hazard of CBC after CPM to be as low as 0.03, relative to UM.^19^ However, that same study found the hazard of death to be significantly lower among patients with CPM, when compared with UM, which is not supported by contemporary literature.^9^, ^11^, ^12^, ^13^ In contrast to this study by Herrinton et al, our study includes over 180 000 patients. While we found a significantly reduced hazard of CBC with CPM, our reported reduction was less striking (HR 0.27 vs 1.0). Therefore, CPM does not eliminate the risk of CBC as suggested by Herrinton et al; although this may be obvious to providers, incomplete protection from CBC should be conveyed to patients who desire CPM when considering the already low risk of CBC in patients without predisposing genetic factors. Our findings may be explained by use of a contemporary cohort with a lower incidence of CBC among patients with UM potentially due to improvements in adjuvant radiation therapy, chemotherapy, and antiestrogen therapy. Consistent with this hypothesis, we also found that higher stage or ER‐positive first primary breast cancers were associated with reduced hazard of CBC. Several studies have previously reported that receipt of adjuvant chemotherapy or antiestrogen therapy is both associated with reduced risk of CBC.^30^, ^31^, ^32^, ^33^ These therapies are most applicable to patients with higher stage tumors or ER‐positive tumors, respectively. Regardless, the reduction in hazard of CBC should be considered in the context of the absolute rates of CBC associated with UM, and the risk of morbidity associated with CPM. For example, the odds of wound and overall 30‐day complications among patients who undergo CPM have been reported to be two times that of patients who undergo UM.^35^ Additionally, patients who undergo breast reconstruction after CPM have increased odds of postoperative complications including infection.^14^, ^36^, ^37^

Our study provides contemporary population‐level analysis of the incidence and hazard ratio of second primary breast cancer in patients who undergo UM or CPM for unilateral ductal carcinoma; however, it is not without limitations. First, it is subject to the usual biases present in large population‐based studies. For example, we are unable to control for tumor properties, such as lymphovascular invasion, and the accuracy of SEER reporting for chemotherapy and radiation therapy is controversial; we did, however, control for tumor stage—a major determinant of therapy. Second, we are unable to account for patient family history or genetic factors such as *BRCA1/2* mutations. These patients have a higher risk of CBC and derive a substantial benefit from CPM.^1^, ^24^ However, it is likely that a higher proportion of patients with *BRCA1/2* mutations comprise the group of CPM patients when compared with the group of UM patients in our analysis; therefore, our analysis may actually *overestimate* the benefit of CPM to patients who do not have *BRCA1/2* mutations. Additionally, we employ a cut‐off of 3 months for the diagnosis of a second primary breast cancer; while this time cut‐off may be material to the categorization of an *ipsilateral* second primary breast cancer versus incomplete resection or recurrence, it likely does not impact the diagnosis of a second primary breast cancer in the contralateral breast. Furthermore, we do not perform a direct comparison between CPM and breast conservation therapy (BCT), which is typically unilateral; we did not perform this comparison in order to avoid the potential confounding of radiation therapy which is a component of BCT.^38^

Overall, our analysis provides a crucial piece of information for clinicians and patients to consider when deciding upon CPM vs UM. While recent studies have been focused on the impact of CPM on patient survival, patient anxiety may not be derived directly from life or death. Instead, patients may be directed by a fear associated with the uncertainty of diagnosis, further surveillance, and the need for future treatment. A more complete understanding of how CPM may impact a patient's clinical course, which includes surveillance and subsequent treatments, may provide patients and physicians with the information they need to make decisions regarding surgical therapy. In addition, systemic therapy which may be initiated after an initial breast cancer diagnosis (eg, antiestrogen therapy) may provide a protective effect to lower the risk of CBC without requiring CPM. That guidelines now recommend up to 15 years of tamoxifen therapy may also provide a longer‐term benefit against CBC. Our findings demonstrate that CPM reduces the hazard of a second primary breast cancer when compared with UM (HR 0.38), but also provides context for this reduction on the basis of cumulative incidence of a second primary breast cancer (10‐year estimated K‐M rate for UM: 4.44%).

## CONFLICTS OF INTEREST

Authors S Agarwal, L Pappas, C Matsen, and J Agarwal declare they have no conflicts of interest.

## AUTHORS’ CONTRIBUTIONS

SA, LP, and JA designed the study. LP performed statistical analysis. SA, LP, CM, and JA interpreted the analysis. SA and JA drafted the manuscript. SA, JA, CM, and LP revised the manuscript.

## Supporting information

Fig S1

## Data Availability

The publicly available SEER database was used for this study. Database access can be obtained at http://seer.cancer.gov/data. The authors are able to share the primary data file used for this study upon request.
