# Supplementary figures and images for: Second primary breast cancer after unilateral mastectomy alone or with contralateral prophylactic mastectomy
Source: Cancer Med. 2020 Sep 12;9(21):8043–52. doi: 10.1002/cam4.3394 (PMC7643660; doi:10.1002/cam4.3394)

Cumulative incidence curves for all incidences in UM and CPM

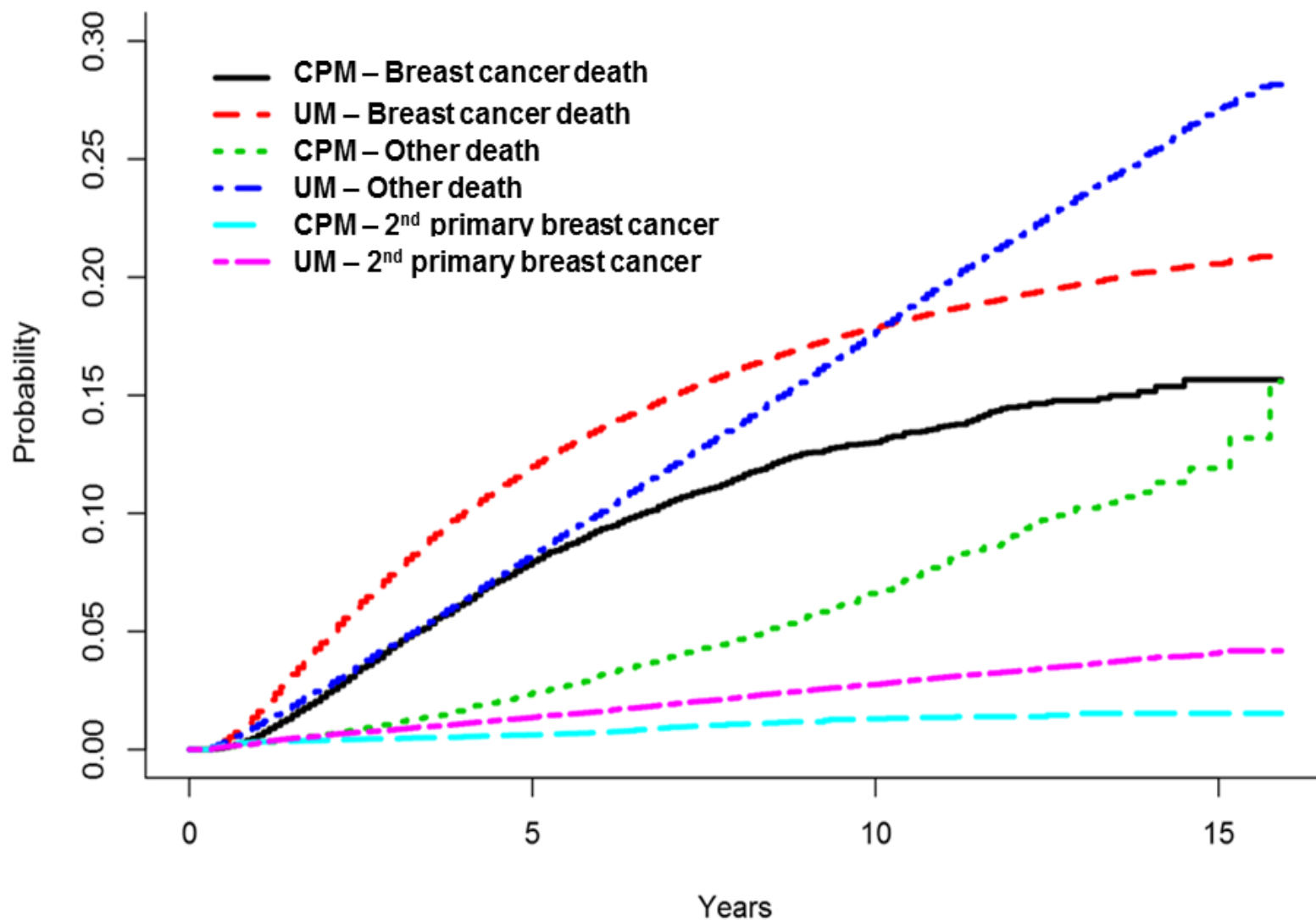

Supplement: Supplementary file 1 — Fig S1 [file CAM4-9-8043-s001.pdf]
